# Genomics and epidemiology of the P.1 SARS-CoV-2 lineage in Manaus, Brazil

**DOI:** 10.1126/science.abh2644

**Published:** 2021-04-14

**Authors:** Nuno R. Faria, Thomas A. Mellan, Charles Whittaker, Ingra M. Claro, Darlan da S. Candido, Swapnil Mishra, Myuki A. E. Crispim, Flavia C. S. Sales, Iwona Hawryluk, John T. McCrone, Ruben J. G. Hulswit, Lucas A. M. Franco, Mariana S. Ramundo, Jaqueline G. de Jesus, Pamela S. Andrade, Thais M. Coletti, Giulia M. Ferreira, Camila A. M. Silva, Erika R. Manuli, Rafael H. M. Pereira, Pedro S. Peixoto, Moritz U. G. Kraemer, Nelson Gaburo, Cecilia da C. Camilo, Henrique Hoeltgebaum, William M. Souza, Esmenia C. Rocha, Leandro M. de Souza, Mariana C. de Pinho, Leonardo J. T. Araujo, Frederico S. V. Malta, Aline B. de Lima, Joice do P. Silva, Danielle A. G. Zauli, Alessandro C. de S. Ferreira, Ricardo P. Schnekenberg, Daniel J. Laydon, Patrick G. T. Walker, Hannah M. Schlter, Ana L. P. dos Santos, Maria S. Vidal, Valentina S. Del Caro, Rosinaldo M. F. Filho, Helem M. dos Santos, Renato S. Aguiar, Jos L. Proena-Modena, Bruce Nelson, James A. Hay, Mlodie Monod, Xenia Miscouridou, Helen Coupland, Raphael Sonabend, Michaela Vollmer, Axel Gandy, Carlos A. Prete, Vitor H. Nascimento, Marc A. Suchard, Thomas A. Bowden, Sergei L. K. Pond, Chieh-Hsi Wu, Oliver Ratmann, Neil M. Ferguson, Christopher Dye, Nick J. Loman, Philippe Lemey, Andrew Rambaut, Nelson A. Fraiji, Maria do P. S. S. Carvalho, Oliver G. Pybus, Seth Flaxman, Samir Bhatt, Ester C. Sabino

**Affiliations:** 1 MRC Centre for Global Infectious Disease Analysis, School of Public Health, Imperial College London, London, UK.; 2 The Abdul Latif Jameel Institute for Disease and Emergency Analytics (J-IDEA), School of Public Health, Imperial College London, London, UK.; 3 Instituto de Medicina Tropical, Faculdade de Medicina da Universidade de So Paulo, So Paulo, Brazil.; 4 Department of Zoology, University of Oxford, Oxford, UK.; 5 Departamento de Molstias Infecciosas e Parasitrias, Faculdade de Medicina da Universidade de So Paulo, So Paulo, Brazil.; 6 Fundao Hospitalar de Hematologia e Hemoterapia, Manaus, Brazil.; 7 Diretoria de Ensino e Pesquisa, Fundao Hospitalar de Hematologia e Hemoterapia, Manaus, Brazil.; 8 Institute of Evolutionary Biology, University of Edinburgh, Edinburgh, UK.; 9 Division of Structural Biology, Wellcome Centre for Human Genetics, University of Oxford, Oxford, UK.; 10 Departamento de Epidemiologia, Faculdade de Sade Pblica da Universidade de So Paulo, Sao Paulo, Brazil.; 11 Laboratrio de Virologia, Instituto de Cincias Biomdicas, Universidade Federal de Uberlndia, Uberlndia, Brazil.; 12 Institute for Applied Economic ResearchIpea, Braslia, Brazil.; 13 Institute of Mathematics and Statistics, University of So Paulo, So Paulo, Brazil.; 14 DB Diagnsticos do Brasil, So Paulo, Brazil.; 15 Department of Mathematics, Imperial College London, London, UK.; 16 Virology Research Centre, Ribeiro Preto Medical School, University of So Paulo, Ribeiro Preto, SP, Brazil.; 17 Laboratory of Quantitative Pathology, Center of Pathology, Adolfo Lutz Institute, So Paulo, Brazil.; 18 Instituto Hermes Pardini, Belo Horizonte, Brazil.; 19 Nuffield Department of Clinical Neurosciences, University of Oxford, Oxford, UK.; 20 CDL Laboratrio Santos e Vidal, Manaus, Brazil.; 21 Departamento de Gentica, Ecologia e Evoluo, Instituto de Cincias Biolgicas, Universidade Federal de Minas Gerais, Belo Horizonte, Brazil.; 22 Laboratory of Emerging Viruses, Department of Genetics, Evolution, Microbiology, and Immunology, Institute of Biology, University of Campinas (UNICAMP), So Paulo, Brazil.; 23 Instituto Nacional de Pesquisas da Amaznia, Manaus, Brazil.; 24 Department of Epidemiology, Harvard T. H. Chan School of Public Health, Boston, MA, USA.; 25 Center for Communicable Disease Dynamics, Harvard T. H. Chan School of Public Health, Boston, MA, USA.; 26 Departamento de Engenharia de Sistemas Eletrnicos, Escola Politcnica da Universidade de So Paulo, So Paulo, Brazil.; 27 Department of Biomathematics, Department of Biostatistics, and Department of Human Genetics, University of California, Los Angeles, CA, USA.; 28 Institute for Genomics and Evolutionary Medicine, Temple University, Philadelphia, PA, USA.; 29 Mathematical Sciences, University of Southampton, Southampton, UK.; 30 Institute for Microbiology and Infection, University of Birmingham, Birmingham, UK.; 31 Department of Microbiology, Immunology and Transplantation, Rega Institute, KU Leuven, Leuven, Belgium.; 32 Diretoria Clnica, Fundao Hospitalar de Hematologia e Hemoterapia do Amazonas, Manaus, Brazil.; 33 Diretoria da Presidncia, Fundao Hospitalar de Hematologia e Hemoterapia do Amazonas, Manaus, Brazil.; 34 Department of Pathobiology and Population Sciences, The Royal Veterinary College, London, UK.; 35 Section of Epidemiology, Department of Public Health, University of Copenhagen, Copenhagen, Denmark.

## Abstract

Cases of severe acute respiratory syndrome coronavirus 2 (SARS-CoV-2) infection in Manaus, Brazil, resurged in late 2020 despite previously high levels of infection. Genome sequencing of viruses sampled in Manaus between November 2020 and January 2021 revealed the emergence and circulation of a novel SARS-CoV-2 variant of concern. Lineage P.1 acquired 17 mutations, including a trio in the spike protein (K417T, E484K, and N501Y) associated with increased binding to the human ACE2 (angiotensin-converting enzyme 2) receptor. Molecular clock analysis shows that P.1 emergence occurred around mid-November 2020 and was preceded by a period of faster molecular evolution. Using a two-category dynamical model that integrates genomic and mortality data, we estimate that P.1 may be 1.7- to 2.4-fold more transmissible and that previous (non-P.1) infection provides 54 to 79% of the protection against infection with P.1 that it provides against non-P.1 lineages. Enhanced global genomic surveillance of variants of concern, which may exhibit increased transmissibility and/or immune evasion, is critical to accelerate pandemic responsiveness.

## Unmitigated spread in Brazil

Despite an extensive network of primary care availability, Brazil has suffered profoundly during the severe acute respiratory syndrome coronavirus 2 (SARS-CoV-2) pandemic. Using daily data from state health offices, Castro *et al.* analyzed the pattern of spread of COVID-19 cases and deaths in the country from February to October 2020. Clusters of deaths before cases became apparent indicated unmitigated spread. SARS-CoV-2 circulated undetected in Brazil for more than a month as it spread north from S o Paulo. In Manaus, transmission reached unprecedented levels after a momentary respite in mid-2020. Faria *et al.* tracked the evolution of a new, more aggressive lineage called P.1, which has 17 mutations, including three (K417T, E484K, and N501Y) in the spike protein. After a period of accelerated evolution, this variant emerged in Brazil during November 2020. Coupled with the emergence of P.1, disease spread was accelerated by stark local inequalities and political upheaval, which compromised a prompt federal response.

*Science*, abh1558 and abh2644, this issue p. 821 and p. 815

## Teaser

A variant lineage of SARS-CoV-2 associated with rapid transmission in Manaus, Brazil, evolved in November 2020 with immune escape characteristics.

Brazil has experienced high mortality during the COVID-19 pandemic, recording >300,000 deaths and >13 million reported cases, as of March 2021. Severe acute respiratory syndrome coronavirus 2 (SARS-CoV-2) infection and disease burden have been highly variable across the country, with the state of Amazonas in north Brazil being the worst-affected region (*1*). Serological surveillance of blood donors in Manaus, the capital city of Amazonas and the largest city in the Amazon region, has suggested >67% cumulative attack rates by October 2020 (*2*). Similar but slightly lower seroprevalences have also been reported for cities in neighboring regions (*3*, *4*). However, the level of previous infection in Manaus was clearly not sufficient to prevent a rapid resurgence in SARS-CoV-2 transmission and mortality there during late 2020 and early 2021 (*5*), which has placed substantial pressure on the citys health care system.

Here, we show that the second wave of infection in Manaus was associated with the emergence and rapid spread of a new SARS-CoV-2 lineage of concern, named lineage P.1. The lineage carries a distinctive constellation of mutations (table S1), including several that have been previously determined to be of virological importance (*6*,*10*) and that are located in the spike protein receptor binding domain (RBD), the region of the virus involved in recognition of the angiotensin-converting enzyme-2 (ACE2) cell surface receptor (*11*). Using genomic data, structure-based mapping of mutations of interest onto the spike protein, and dynamical epidemiology modeling of genomic and mortality data, we investigated the emergence of the P.1 lineage and explored epidemiological explanations for the resurgence of COVID-19 in Manaus.

## Identification and nomenclature of the P.1 lineage in Manaus

In late 2020, two SARS-CoV-2 lineages of concern were discovered through genomic surveillance, both characterized by sets of notable mutations: lineage B.1.351, first reported in South Africa (*12*), and lineage B.1.1.7, detected in the UK (*13*)*.* Both variants have transmitted rapidly in the countries where they were discovered and spread to other regions (*14*, *15*). Analyses indicate that B.1.1.7 has higher transmissibility and causes more severe illness as compared with those of previously circulating lineages in the UK (*1*, *16*, *17*).

**Fig. 1:**
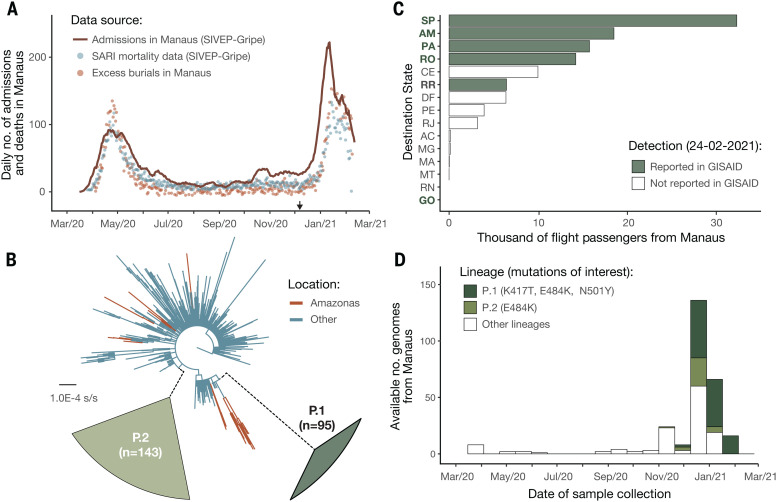
SARS-CoV-2 epidemiological, diagnostic, genomic, and mobility data from Manaus. (**A**) Dark solid line shows the 7-day rolling average of the COVID-19 confirmed and suspected daily time series of hospitalizations in Manaus. Admissions in Manaus are from Fundao de Vigilncia em Sade do Amazonas (*66*). Green dots indicate daily severe acute respiratory mortality records from the SIVEP-Gripe (Sistema de Informao de Vigilncia Epidemiolgica da Gripe) database (*67*). Red dots indicate excess burial records based on data from Manaus Mayors office for comparison (supplementary materials, materials and methods). The arrow indicates 6 December 2020, the date of the first P.1 case identified in Manaus by our study. (**B**) Maximum likelihood tree (*n* = 962 viral genomes) with B.1.1.28, P.1, and P.2 sequences, with collapsed views of P.1 and P.2 clusters and highlighting other sequences from Amazonas state, Brazil. Ancestral branches leading to P.1 and P.2 are shown as dashed lines. A more detailed phylogeny is available in fig. S3. Scale bar is shown in units of nucleotide substitutions per site (s/s). (**C**) Number of air travel passengers from Manaus to all states in Brazil was obtained from National Civil Aviation Agency of Brazil (www.gov.br/anac). The ISO 3166-2:BR codes of the states with genomic reports of P.1 [GISAID (*68*), as of 24 February 2021], are shown in bold. An updated list of GISAID genomes and reports of P.1 worldwide is available at https://cov-lineages.org/global_report_P.1.html. (**D**) Number of genome sequences from Manaus belonging to lineages of interest (supplementary materials, materials and methods). Spike mutations of interest are denoted.

After a rapid increase in hospitalizations in Manaus caused by severe acute respiratory infection (SARI) in December 2020 (Fig. 1A), we focused ongoing SARS-CoV-2 genomic surveillance (*2*, *18*,*22*) on recently collected samples from the city (supplementary materials, materials and methods, and table S2). Before this, only seven SARS-CoV-2 genome sequences from Amazonas were publicly available (SARS-CoV-2 was first detected in Manaus on 13 March 2020) (*19*, *23*). We sequenced SARS-CoV-2 genomes from 184 samples from patients seeking COVID-19 testing in two diagnostic laboratories in Manaus between November and December 2020, using the ARTIC V3 multiplexed amplicon scheme (*24*) and the MinION sequencing platform. Because partial genome sequences can provide useful epidemiological information, particularly regarding virus genetic diversity and lineage composition (*25*), we harnessed information from partial (*n* = 41 viral sequences, 25 to 75% genome coverage), as well as near-complete (*n* = 95 viral sequences, 75 to 95%) and complete (*n* = 48 viral sequences, 95%) sequences from Manaus (figs. S1 to S4), together with other available and published genomes from Brazil for context. Viral lineages were classified by using the Pangolin (*26*) software tool (http://pangolin.cog-uk.io), nextclade (https://clades.nextstrain.org), and standard phylogenetic analysis using complete reference genomes.

Our early data indicated the presence of a novel SARS-CoV-2 lineage in Manaus that contained 17 amino acid changes (including 10 in the spike protein), three deletions, four synonymous mutations, and a fourbase-pair nucleotide insertion compared with the most closely related available sequence (GISAID ID: EPI_ISL_722052) (Fig. 1B; lineage-defining mutations can be found in table S1) (*27*). This lineage was given a new designation, P.1, on the basis that (i) it is phylogenetically and genetically distinct from ancestral viruses, (ii) associated with rapid spread in a new area, and (iii) carries a constellation of mutations that may have phenotypic relevance (*26*). Phylogenetic analysis indicated that P.1and another lineage, P.2 (*19*)were descendants of lineage B.1.1.28 that was first detected in Brazil in early March 2020 (Fig. 1B). Our preliminary results were shared with local teams on 10 January 2021 and published online on 12 January 2021 (*27*). Concurrently, cases of SARS-CoV-2 P.1 infection were reported in Japan in travelers from Amazonas (*28*). As of 24 February 2021, P.1 had been confirmed in six Brazilian states, which in total received >92,000 air passengers from Manaus in November 2020 (Fig. 1C). Genomic surveillance first detected lineage P.1 on 6 December 2020 (Fig. 1A), after which the frequency of P.1 relative to other lineages increased rapidly in the tested samples from Manaus (Fig. 1D; lineage frequency information can be found in fig. S5). Retrospective genome sequencing might be able to recover earlier P.1 genomes. Between 2 November 2020 and 9 January 2021, we observed 7137 SARI cases and 3144 SARI deaths in Manaus (Fig. 1A). We generated a total of 182 SARS-CoV-2 sequences from Manaus during this period. This corresponds to one genome for each 39 SARI cases in Manaus, and this ratio is >100-fold higher as compared with the average number of shared genomes per reported case during the same period in Brazil.

## Dating the emergence of the P.1 lineage

We used molecular-clock phylogenetics to understand the emergence and evolution of lineage P.1 (*25*). We first regressed root-to-tip genetic distances against sequence sampling dates (*29*) for the P.1, P.2, and B.1.1.28 lineages separately (figs. S6 to S8). This exploratory analysis revealed similar evolutionary rates within each lineage but greater root-to-tip distances for P.1 compared with B.1.1.28 (fig. S8), suggesting that the emergence of P.1 was preceded by a period of faster molecular evolution. The B.1.1.7 lineage exhibits similar evolutionary characteristics (*13*), which was hypothesized to have occurred in a chronically infected or immunocompromised patient (*30*, *31*).

**Fig. 2:**
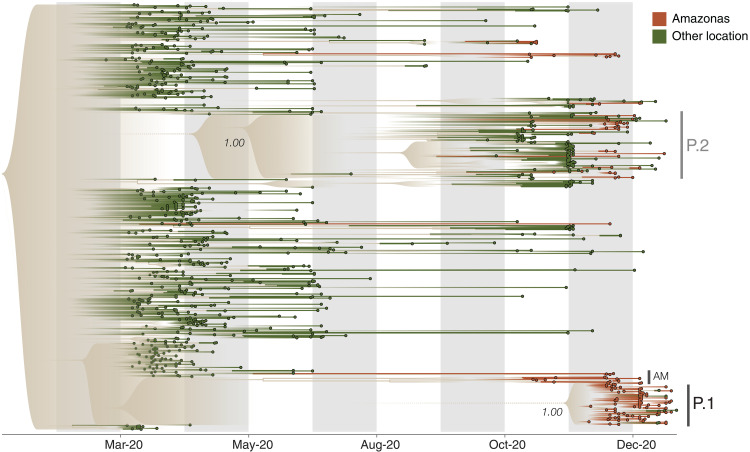
Visualization of the time-calibrated maximum clade credibility tree reconstruction for B.1.1.28, P.1, and P.2 lineages in Brazil. Terminal branches and tips of Amazonas state are colored in brown, and those from other locations are colored in green (*n* = 962 viral genomes). Nodes with posterior probabilities of <0.5 have been collapsed into polytomies, and their range of divergence dates are illustrated as shaded expanses.

To date the emergence of P.1, while accounting for a faster evolutionary rate along its ancestral branch, we used a local molecular clock model (*32*) with a flexible nonparametric demographic tree prior (*33*). Using this approach, we estimated the date of the common ancestor of the P.1 lineage to be around 15 November 2020 [median, 95% Bayesian credible interval (BCI), 6 October to 24 November 2020; mean, 9 November 2020] (fig. S9). This is only 3 to 4 weeks before the resurgence in SARS-CoV-2 confirmed cases in Manaus (Figs. 1A and 2 and fig. S9). The P.1 sequences formed a single well-supported group (posterior probability = 1.00) that clustered most closely with B.1.1.28 sequences from Manaus (Fig. 2, AM), suggesting that P.1 emerged there. The earliest P.1 samples were detected in Manaus (*34*). The first known travel-related cases were detected in Japan (*28*) and So Paulo (table S3) and were both linked to travel from Manaus. Furthermore, the local clock model statistically confirmed a higher evolutionary rate for the branch immediately ancestral to lineage P.1 compared with lineage B.1.1.28 as a whole [Bayes factor (BF) = 6.04].

Our data indicate multiple introductions of the P.1 lineage from Amazonas to Brazils southeastern states (Fig. 2). We also detected seven small well-supported clusters of P.2 sequences from Amazonas (two to six sequences, posterior probability = 1.00). Virus exchange between Amazonas state and the urban metropolises in southeast Brazil largely follows patterns of national air travel mobility (Fig. 1D and fig. S10).

## Infection with P.1 and sample viral loads

We analyzed all quantitative reverse transcription polymerase chain reaction (RT-PCR) SARS-CoV-2positive results from a laboratory that has provided testing in Manaus since May 2020 (Fig. 1A and data file S1), with the aim of exploring trends in sample quantitative RT-PCR cycle threshold (Ct) values, which are inversely related to sample virus loads and transmissibility (*35*). By focusing on data from a single laboratory, we reduced instrument and process variation that can affect Ct measurements.

**Fig. 3:**
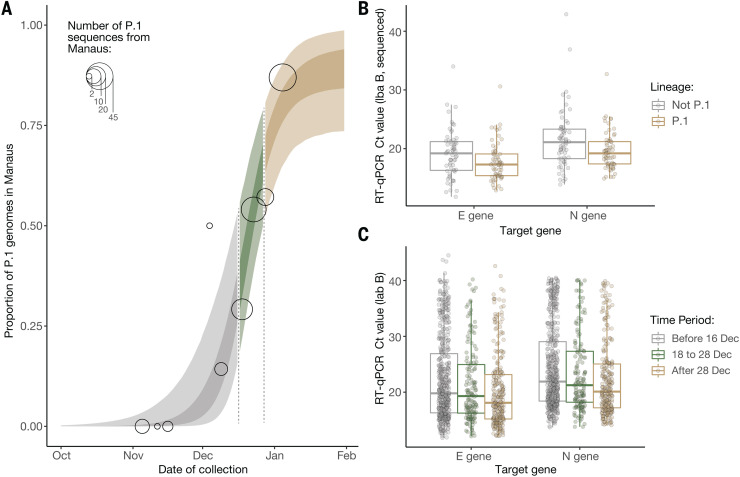
Temporal variation in the proportion of sequenced genomes belonging to P.1, and trends in quantitative RT-PCR Ct values for COVID-19 infections in Manaus. (**A**) Logistic function fitting to the proportion of genomes in sequenced infections that have been classified as P.1 (black circles, size indicating number of infections sequenced), divided up into time periods when the predicted proportion of infections that are due to P.1 is <1/3 (light brown), between 1/3 and 2/3 (green), and greater than 2/3 (gray). For the model fit, the darker ribbon indicates the 50% credible interval, and the lighter ribbon indicates the 95% credible interval. For the data points, the gray thick line is the 50% exact binomial CI, and the thinner line is the 95% exact binomial CI. (**B**) Ct values for genes E and N in a sample of symptomatic cases presenting for testing at a health care facility in Manaus (laboratory A), stratified according to the period defined in (A) in which the oropharyngeal and nasal swab collections occurred. (**C**) Ct values for genes E and N in a subsample of 184 infections included in (B) that had their genomes sequenced (dataset A).

We analyzed a set of quantitative RT-PCR positive cases for which virus genome sequencing and lineage classification had been undertaken (*n* = 147 samples). Using a logistic function (Fig. 3A), we found that the fraction of samples classified as P.1 increased from 0 to 87% in around 7 weeks (table S4), quantifying the trend shown in Fig. 1C. We found a small but statistically significant association between P.1 infection and lower Ct values, for both the *E* gene (lognormal regression, *P* = 0.029, *n* = 128 samples, 65 of which were P.1) and *N* gene (*P* = 0.01, *n* = 129 samples, 65 of which were P.1), with Ct values lowered by 1.43 [0.17 to 2.60, 95% confidence interval (CI)] and 1.91 (0.49 to 3.23) cycles in the P.1 lineage on average, respectively (Fig. 3B).

Using a larger sample of 942 Ct values (including an additional 795 samples for which no lineage information was available), we investigated Ct values across three time periods characterized by increasing P.1 relative abundance. Average Ct values for both the *E* and *N* genes declined through time, as both case numbers and the fraction of P.1 infections increased, with Ct values significantly lower in period 3 as compared with period 1 (*E* gene, *P* = 0.12 and *P* < 0.001 for comparison of time periods 2 and 3 to period 1; *N* gene, *P* = 0.14 and *P* < 0.001, respectively) (Fig. 3C). Analyses of Ct values for samples from a different laboratory, also based in Manaus, showed similarly significant declines between the first and third time periods defined here (*P* < 0.0001 for both *E* and *N* genes) (fig. S11 and data file S3).

However, population-level Ct distributions are sensitive to changes in the average time since infection when samples are taken, so that median Ct values can decrease during epidemic growth periods and increase during epidemic decline (*36*). To account for this effect, we assessed the association between P.1 infection and Ct levels while controlling for the delay between symptom onset and sample collection. Statistical significance was lost for both data sets (E gene, *P* = 0.15, *n* = 42 samples, 22 of which were P.1; N gene, *P* = 0.12, *n* = 42 samples, 22 of which were P.1). Owing to this confounding factor, we cannot distinguish whether P.1 infection is associated with increased viral loads (*37*) or a longer duration of infection (*38*).

## Mathematical modeling of lineage P.1 epidemiological characteristics

We next explored epidemiological scenarios that might explain the recent resurgence of transmission in Manaus (*39*). To do this, we extended a semimechanistic Bayesian model of SARS-CoV-2 transmissibility and mortality (*40*,*42*) to include two categories of virus (P.1 and non-P.1) and to account for infection severity, transmissibility, and propensity for reinfection to vary between the categories. It also integrates information on the timing of P.1 emergence in Manaus using our molecular clock results (Fig. 2). The model explicitly incorporates waning of immune protection after infection, parameterized on the basis of dynamics observed in recent studies (*16*, *43*), to explore the competing hypothesis that waning of prior immunity might explain the observed resurgence (*42*). We used the model to evaluate the statistical support that P.1 possesses altered epidemiological characteristics compared with local non-P.1 lineages. Epidemiological model details and sensitivity analyses (tables S5 to S10) can be found in the supplementary materials. The model is fitted to both COVID-19 mortality data [with a correction for systematic reporting delays (*44*, *45*)] and the estimated increase through time in the proportion of infections due to P.1 derived from genomic data (table S4). We assumed that within-category immunity wanes over time (50% wane within a year, although sensitivity analyses varying the rapidity of waning are presented in table S7) and that cross-immunity (the degree to which previous infection with a virus belonging to one category protects against subsequent infection with the other) is symmetric between categories.

**Fig. 4:**
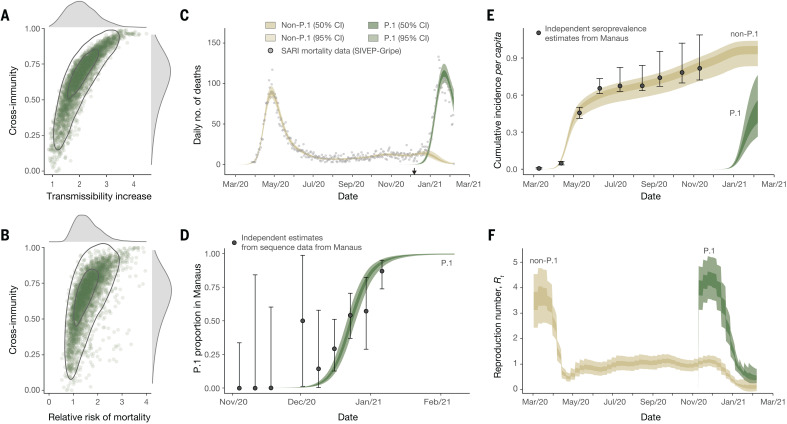
Estimates of the epidemiological characteristics of P.1 inferred from a multicategory Bayesian transmission model fitted to data from Manaus, Brazil. (**A**) Joint posterior distribution of the cross-immunity and transmissibility increase inferred through fitting the model to mortality and genomic data. Gray contours indicate posterior density intervals ranging from the 95 and 50% isoclines. Marginal posterior distributions for each parameter shown along each axis. (**B**) As for (A), but showing the joint-posterior distribution of cross-immunity and the inferred relative risk of mortality in the period after emergence of P.1 compared with the period prior. (**C**) Daily incidence of COVID-19 mortality. Points indicate severe acute respiratory mortality records from the SIVEP-Gripe database (*67*, *69*). Brown and green ribbons indicate model fit for COVID-19 mortality incidence, disaggregated by mortality attributable to non-P.1 lineages (brown) and the P.1 lineage (green). (**D**) Estimate of the proportion of P.1 infections through time in Manaus. Black data points with error bars are the empirical proportion observed in genomically sequenced cases (Fig. 3A), and green ribbons (dark = 50% BCI, light = 95% BCI) are the model fit to the data. (**E**) Estimated cumulative infection incidence for the P.1 and non-P.1 categories. Black data points with error bars are reversion-corrected estimates of seroprevalence from blood donors in Manaus (*2*). Colored ribbons are the model predictions of cumulative infection incidence for non-P.1 lineages (brown) and P.1 lineages (green). These points are shown for reference only and were not used to fit the model. (**F**) Bayesian posterior estimates of trends in reproduction number *R*_t_ for the P.1 and non-P.1 categories.

Our results suggest that the epidemiological characteristics of P.1 are different from those of previously circulating local SARS-CoV-2 lineages, but the results also highlight substantial uncertainty in the extent and nature of this difference. Plausible values of transmissibility and cross-immunity exist in a limited area but are correlated (Fig. 4A, with the extent of immune evasion defined as 1 minus the inferred cross-immunity). This is expected because in the model, a higher degree of cross-immunity means that greater transmissibility of P.1 is required to generate a second epidemic. Within this plausible region of parameter space, P.1 can be between 1.7 and 2.4 times more transmissible (50% BCI, 2.0 median, with a 99% posterior probability of being >1) than local non-P1 lineages and can evade 21 to 46% (50% BCI, 32% median, with a 95% posterior probability of being able to evade at least 10%) of protective immunity elicited by previous infection with non-P.1 lineages, corresponding to 54 to 79% (50% BCI, 68% median) cross-immunity (Fig. 4A). The joint-posterior distribution is inconsistent with a combination of highly increased transmissibility and low cross-immunity and, conversely, also with near-complete cross-immunity but only a small increase in transmissibility (Fig. 4A). Moreover, our results further show that natural immunity waning alone is unlikely to explain the observed dynamics in Manaus, with support for P.1 possessing altered epidemiological characteristics robust to a range of values assumed for the date of the lineages emergence and the rate of natural immunity waning (tables S5 and S7). We caution that these results are not generalizable to other settings; more detailed and direct data are needed to identify the exact degree and nature of the changes to the epidemiological characteristics of P.1 compared with previously circulating lineages.

We estimate that infections are 1.2 to 1.9 times more likely (50% BCI, median 1.5, 90% posterior probability of being >1) to result in mortality in the period after the emergence of P.1, compared with before, although posterior estimates of this relative risk are also correlated with inferred cross-immunity (Fig. 4B). More broadly, the recent epidemic in Manaus has strained the citys health care system, leading to inadequate access to medical care (*46*). We therefore cannot determine whether the estimated increase in relative mortality risk is due to P.1 infection, stresses on the Manaus health care system, or both. Detailed clinical investigations of P.1 infections are needed. Our model makes the assumption of a homogeneously mixed population and therefore ignores heterogeneities in contact patterns (differences in private versus public hospitals are provided in fig. S13). This is an important area for future research. The model fits observed time series data from Manaus on COVID-19 mortality (Fig. 4C) and the relative frequency of P.1 infections (Fig. 4D) and also captures previously estimated trends in cumulative seropositivity in the city (Fig. 4E). We estimate the reproduction number (*R*_t_) on 7 February 2021 to be 0.1 (median, 50% BCI, 0.04 to 0.2) for non-P.1 and 0.5 (median, 50% BCI, 0.4 to 0.6) for P.1 (Fig. 4F).

## Characterization and adaptation of a constellation of spike protein mutations

Lineage P.1 contains 10 lineage-defining amino acid mutations in the virus spike protein (L18F, T20N, P26S, D138Y, R190S, K417T, E484K, N501Y, H655Y, and T1027I) compared with its immediate ancestor (B.1.1.28). In addition to the possible increase in the rate of molecular evolution during the emergence of P.1, we found by use of molecular selection analyses (*47*) evidence that eight of these 10 mutations are under diversifying positive selection (table S1 and fig. S14). (Single-letter abbreviations for the amino acid residues are as follows: A, Ala; C, Cys; D, Asp; E, Glu; F, Phe; G, Gly; H, His; I, Ile; K, Lys; L, Leu; M, Met; N, Asn; P, Pro; Q, Gln; R, Arg; S, Ser; T, Thr; V, Val; W, Trp; and Y, Tyr. In the mutants, other amino acids were substituted at certain locations; for example, K417T indicates that lysine at position 417 was replaced by threonine.)

Three key mutations present in P.1N501Y, K417T, and E484Kare in the spike protein RBD. The former two interact with human ACE2 (hACE2) (*11*), whereas E484K is located in a loop region outside the direct hACE2 interface (fig. S14). The same three residues are mutated with the B.1.351 variant of concern, and N501Y is also present in the B.1.1.7 lineage. The independent emergence of the same constellation of mutations in geographically distinct lineages indicates a process of convergent molecular adaptation. Similar to SARS-CoV-1 (*48*,*50*), mutations in the RBD may increase affinity of the virus for host ACE2 and consequently influence host cell entry and virus transmission. Recent molecular analysis of B.1.351 (*51*) indicates that the three P.1 RBD mutations may similarly enhance hACE2 engagement, providing a plausible hypothesis for an increase in transmissibility of the P.1 lineage. Moreover, E484K is associated with reduced antibody neutralization (*6*, *9*, *52*, *53*). RBD-presented epitopes account for ~90% of the neutralizing activity of sera from individuals previously infected with SARS-CoV-2 (*54*); thus, tighter binding of P.1 viruses to hACE2 may further reduce the effectiveness of neutralizing antibodies.

## Conclusion

We show that P.1 most likely emerged in Manaus in mid-November, where high attack rates have been previously reported. High rates of mutation accumulation over short time periods have been reported in chronically infected or immunocompromised patients (*13*). Given a sustained generalized epidemic in Manaus, we believe that this is a potential scenario for P.1 emergence. Genomic surveillance and early data sharing by teams worldwide have led to the rapid detection and characterization of SARS-CoV-2 and new variants of concern (VOCs) (*25*), yet such surveillance is still limited in many settings. The P.1 lineage is spreading rapidly across Brazil (*55*), and this lineage has now been detected in >36 countries (*56*). But existing virus genome sampling strategies are often inadequate for determining the true extent of VOCs in Brazil, and more detailed data are needed to address the impact of different epidemiological and evolutionary processes in their emergence. Sustainable genomic surveillance efforts to track variant frequency [for example, (*57*,*59*)] coupled with analytical tools to quantify lineage dynamics [for example, (*60*, *61*)] and anonymized epidemiological surveillance data (*62*, *63*) could enable enhanced real-time surveillance of VOCs worldwide. Studies to evaluate real-world vaccine efficacy in response to P.1 are urgently needed. Neutralization titers represent only one component of the elicited response to vaccines, and minimal reduction of neutralization titers relative to earlier circulating strains is not uncommon. Until an equitable allocation and access to effective vaccines is available to all, nonpharmaceutical interventions should continue to play an important role in reducing the emergence of new variants.

## Supplementary Materials

science.sciencemag.org/content/372/6544/815/suppl/DC1 Materials and Methods Supplementary Text Figs. S1 to S16 Tables S1 to S10 References (*70*,*102*) Data Files S1 to S6 MDAR Reproducibility Checklist
